# Mechanisms of ivermectin-induced wound healing

**DOI:** 10.1186/s12917-020-02612-z

**Published:** 2020-10-20

**Authors:** Daniel Kwesi Sia, Kwesi Boadu Mensah, Tony Opoku-Agyemang, Raphael D. Folitse, David Obiri Darko

**Affiliations:** 1 Department of Pharmacology, College of Health Sciences, Kwame Nkrumah University of Science and Technology, Kumasi, Ghana; 2 School of Veterinary Medicine, College of Health Sciences, Kwame Nkrumah University of Science and Technology, Kumasi, Ghana

**Keywords:** Ivermectin, Hydroxyproline, TGF-β 1, VEGF, Cytokines, Growth factors, Eicosanoids

## Abstract

**Background:**

Wounds cause structural and functional discontinuity of an organ. Wound healing, therefore, seeks to re-establish the normal morphology and functionality through intertwined stages of hemostasis, inflammation, proliferation, and tissue remodelling. Ivermectin, a macrolide, has been used as an endectoparasiticide in human and veterinary medicine practice for decades. Here, we show that ivermectin exhibits wounding healing activity by mechanisms independent of its well-known antiparasitic activity. This study aimed to evaluate the wound healing property of ivermectin cream using histochemistry and enzyme-linked immunosorbent assay techniques.

**Results:**

Non-irritant dose of ivermectin cream (0.03–1%) decreased wound macroscopic indices such as exudation, edge edema, hyperemia, and granulation tissue deposition by day 9 compared to day 13 for the vehicle-treated group. This corresponded with a statistically significant wound contraction rate, hydroxyproline deposition, and a decreased time to heal rate. The levels of growth factors TGF-β1 and VEGF were significantly elevated on day 7 but decreased on day 21. This corresponded with changes in cytokines (IL-1α, IL-4, IL-10, and TNF-α) and eicosanoids (LTB4, PGE_2_, and PGD_2_) levels on days 7 and 21._._ Interestingly, low doses of ivermectin cream (0.03–0.1%) induced wound healing with minimal scarring compared to higher doses of the cream and the positive control, Silver Sulfadiazine.

**Conclusion:**

Ivermectin promotes wound healing partly through modulation of the inflammatory process and the levels of Transforming Growth Factor-Beta 1 and Vascular Endothelial Growth Factor. Low doses of ivermectin cream have the potential to be used in treating wounds with minimal scar tissue formation.

## Background

A wound is a break in the structural continuity regarding the morphology and the functionality of an organ [1]. Wound healing is, therefore, a progression of an intricate biochemical and physiological cascade that reestablishes the integral anatomy and functionality of the injured tissue [2]. Wound repair is a critical yet entangled process in mammals, with many different features represented by successive yet interwoven phases; hemostatic, inflammatory, proliferative, and maturation phases [3]. Immediately after injury, the disrupted fibers activate platelet aggregation, resulting in degranulation and the release of clotting, chemotactic, and growth factors to initiate hemostasis [4]. Fibronectin, thrombin, and their derivatives from platelets interface with collagen leading to the release of growth factors and cytokines. A clot formed as a result of tissue damage serves as an adhesion site for cells such as fibroblast, neutrophils, endothelial cells, and monocytes that migrate to the wounded site [2].

Hydroxyproline, an integral component of collagen, serves as a precursor of tissue fibers; MPO, an enzyme mainly released by neutrophils and monocytes, combats microbial invasion during injury and trigger inflammation [5]. Eicosanoids such as Prostaglandin E_2_ (PGE_2_), Leukotriene B_4_ (LTB_4_) tend to enhance inflammation whereas Prostaglandin D_2_ (PGD_2_) shows anti-inflammatory actions during wound healing [6, 7]. Many cytokines and growth factors are implicated in the wound healing cascade. They exhibit either pro-inflammatory or anti-inflammatory activity [8]. The interleukins; IL-1α, IL-1β, IL-4, and TNF-α are pro-inflammatory whilst IL-10 is anti-inflammatory during the wound healing process [5]. TGF-β1 and VEGF are the two major growth factors that play a very significant role in the restoration of tissue function and integrity [9].

Ivermectin is a macrolide belonging to the avermectin group which first received a wide application in human and veterinary medicine practice about three decades ago [10]. Ivermectin was found to be so useful as an antiparasitic agent in many species and, it can be administered topically, orally, or parenterally [11]. Ivermectin eliminates parasites by inducing hyperpolarization through binding to the parasite-specific gamma-aminobutyric acid (GABA)-gated chloride ions and glutamate-gated ion channels; dampening transmission of electrical impulses resulting in paralysis and death [11, 12]. Ivermectin is used in the field to treat mite infestation and maggot-infested wounds. Its selective toxicity is because susceptible parasite GABA receptors are distributed in the Peripheral Nervous System whereas the host GABA receptors are distributed in the CNS [11, 12].

It was observed to rid sore-skin and open wounds of mites and flystrike maggots; as such facilitating wound healing [11, 13]. The need to study the wound healing potential of ivermectin arose from reviewing several published works on the drug and uncovering its anti-inflammatory and antimicrobial activity [3, 14]. We hypothesized that ivermectin promotes wound healing partly through mechanisms independent of its antiparasitic activity. The aim of this study, therefore, was to evaluate the wound healing property of ivermectin in Sprague Dawley rats.

## Results

### Acute dermatotoxicity study

The Draize dermal irritation test indicated that all the three topical doses of ivermectin cream (1–10% (w/w)) caused no erythema, edema, or skin eruption. The Primary Irritation Index was estimated to be less than 1 (PII < 1). This means that the ivermectin cream was nonirritant and nontoxic to the skin up to 10% (w/w).

### Effects of ivermectin on macroscopic wound healing indices

#### Wound exudation index

None of the study groups exhibited moderate or severe exudation. However, all ivermectin treated wounds were less exudative than the control wounds. There was an inverse relationship between ivermectin dose and wound exudation. As such, the lowest dose of ivermectin was most effective in reducing exudation (Table 1).

**Table 1:** Effects of Ivermectin cream on Macroscopic Wound Healing Indices

WOUND HEALING INDICES	CONTROL	SILVER SULFADIAZINE	IVERMECTIN CREAM (% W/W)			
			1	0.3	0.1	0.03
Exudation Index	0.30 ± 0.18	0.25 ± 0.16**	0.25 ± 0.16**	0.13 ± 0.12***	0.13 ± 0.12***	0.00 ± 0.00****
Edge Edema Index	2.63 ± 0.96	2.38 ± 0.90*	2.13 ± 0.81**	1.88 ± 0.87**	1.63 ± 0.80***	1.25 ± 0.77***
Hyperaemia Index	2.00 ± 0.94	2.00 ± 0.94*	1.75 ± 0.96*	1.88 ± 0.93**	1.63 ± 0.96****	1.63 ± 0.96****
Granulation Tissue Index	4.00 ± 0.60	2.38 ± 0.92*	2.25 ± 0.86*	1.88 ± 0.87**	1.63 ± 0.80***	1.25 ± 0.77****

Sprague Dawley rats were anaesthetised as described in the methods. Excision wounds were created and evaluated systematically on days 0, 2, 5, 7, 9, 13, 15 and 21 for macroscopic wound healing indices (0 = Absent, 1–2 = Mild, 3–4 = Moderate, 5–6 = Severe). Data is present as mean ± SEM of wound exudation indices. Statistical analysis is by One-way ANOVA. * means *p* < 0.05, ** *p* < 0.01, *** *p* < 0.001, **** *p* < 0.0001 when compared to control using Bonferroni's post hoc test

#### Wound edge edema index

All wounds, on day 0, had swollen edges after a few hours of in-depth cutaneous wounding. On day 9, the edge edema of all ivermectin treated wounds had resolved. Wound edge edema of the control animals did not resolve completely until day13 (Table 1).

#### Wound hyperemia index

The sign of excessive blood flow to the wound site such as hyperemia was indexed. All wounds were highly hyperemic on day 0 to 2 post-wounding and began to resolve on the subsequent days. All ivermectin treated wounds were less hyperemic than control wounds. On day 9, hyperemia had completely resolved in all groups. Low doses of ivermectin (0.03–0.10% (w/w)) enhanced resolution of hyperemia faster (by day 7) than the high doses and the positive control of Silver Sulfadiazine (Table 1).

#### Granulation tissue index

The granulation tissue deposition was high in all groups within 24 h post-trauma. The granulation tissue receded completely on day 7 for 0.03% (w/w) ivermectin-treated wounds followed by complete granulation tissue recession on day 9 for 0.10 and 0.30% (w/w) ivermectin-treated wounds. Except for the control group whose wounds still had evidence of granulation, all other wounds had no evidence of tissue granulation after day 13 (Table 1).

### Wound morphometry: cutaneous wound contraction evaluation

**Fig. 1:**
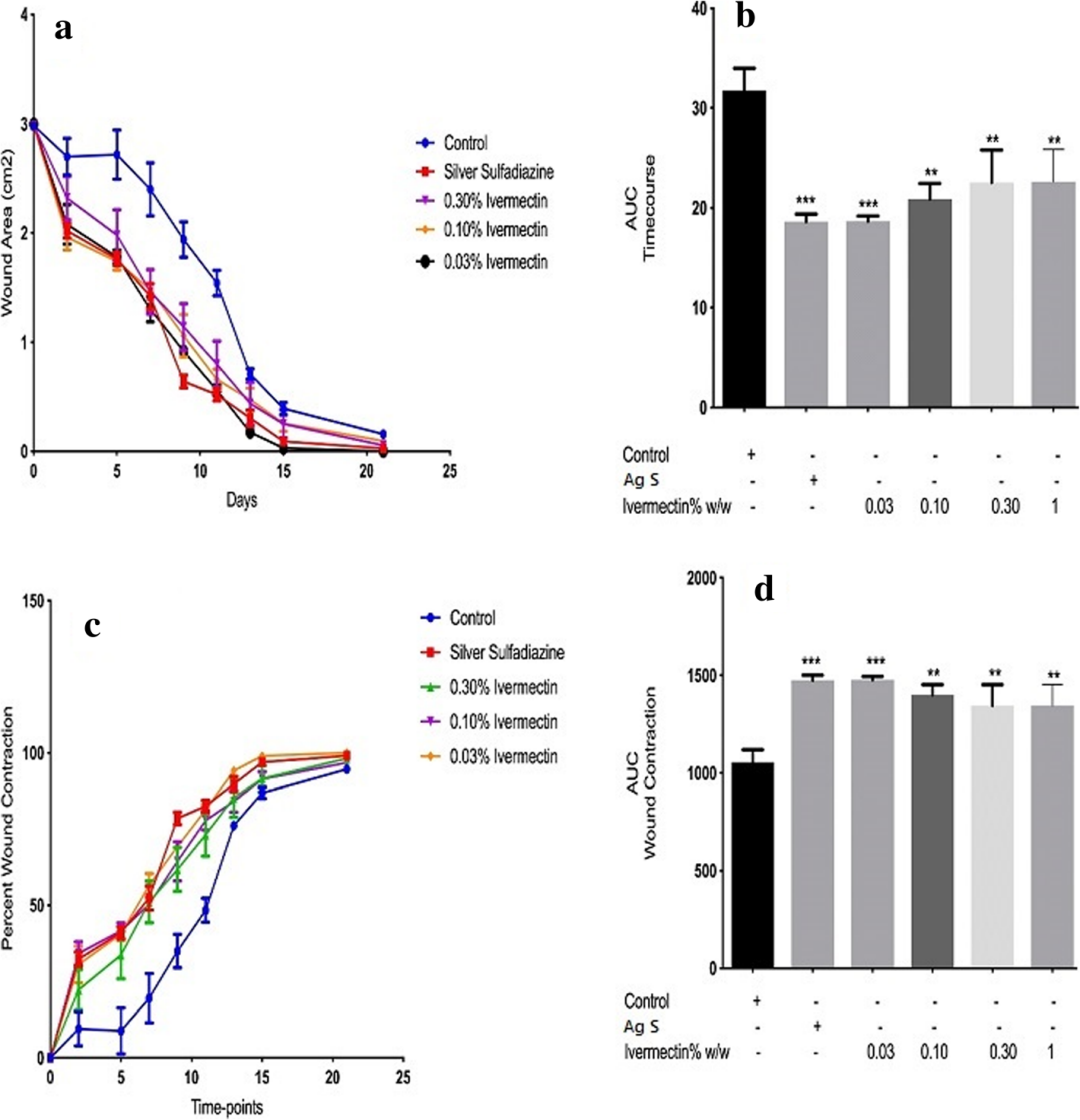
Morphometric evaluation of the effects of ivermectin cream on cutaneous wound contraction. The time-course of the healing process (**a**) with the corresponding Areas Under Curve (AUC) (**b**); Wound Contraction rate (WC) expressed in percentages (**c**) with the corresponding AUCs (**d**) are as presented. Statistical analysis for each AUCs is by One-Way ANOVA. ** means *p* < 0.01 and *** means *p* < 0.001 when compared to the vehicle (naïve) control group

The time-course curve (Fig. 1a) and its corresponding column graphs (AUC) in Fig. 1b, as well as the wound closure per cent curve and AUCs in Fig. 1c & d showed the “time-to-heal” progression and the percentage of wound closure respectively. Between day 5 and day 10, all ivermectin-treated wounds recorded the approximate mean surface areas and mean percentage wound closure of 160 ± 0.91 mm^2^ at 48.5 ± 1.2% (day 5), 140 ± 0.97 mm^2^ at 59 ± 1.3% (day 7), 100 ± 0.995 mm^2^ at 68 ± 1.0% (day 10) shown in Fig. 1a & c. After day 10, the lowest dose of the ivermectin-treated wounds had fairly the most accelerated wound healing (***p < 0.001). The 0.03% (w/w) ivermectin-treated wounds showed, on day 21, a nearly perfect wound closure shown in Figure S1.

### Wound histological analysis on day 21

**Fig. 2:**
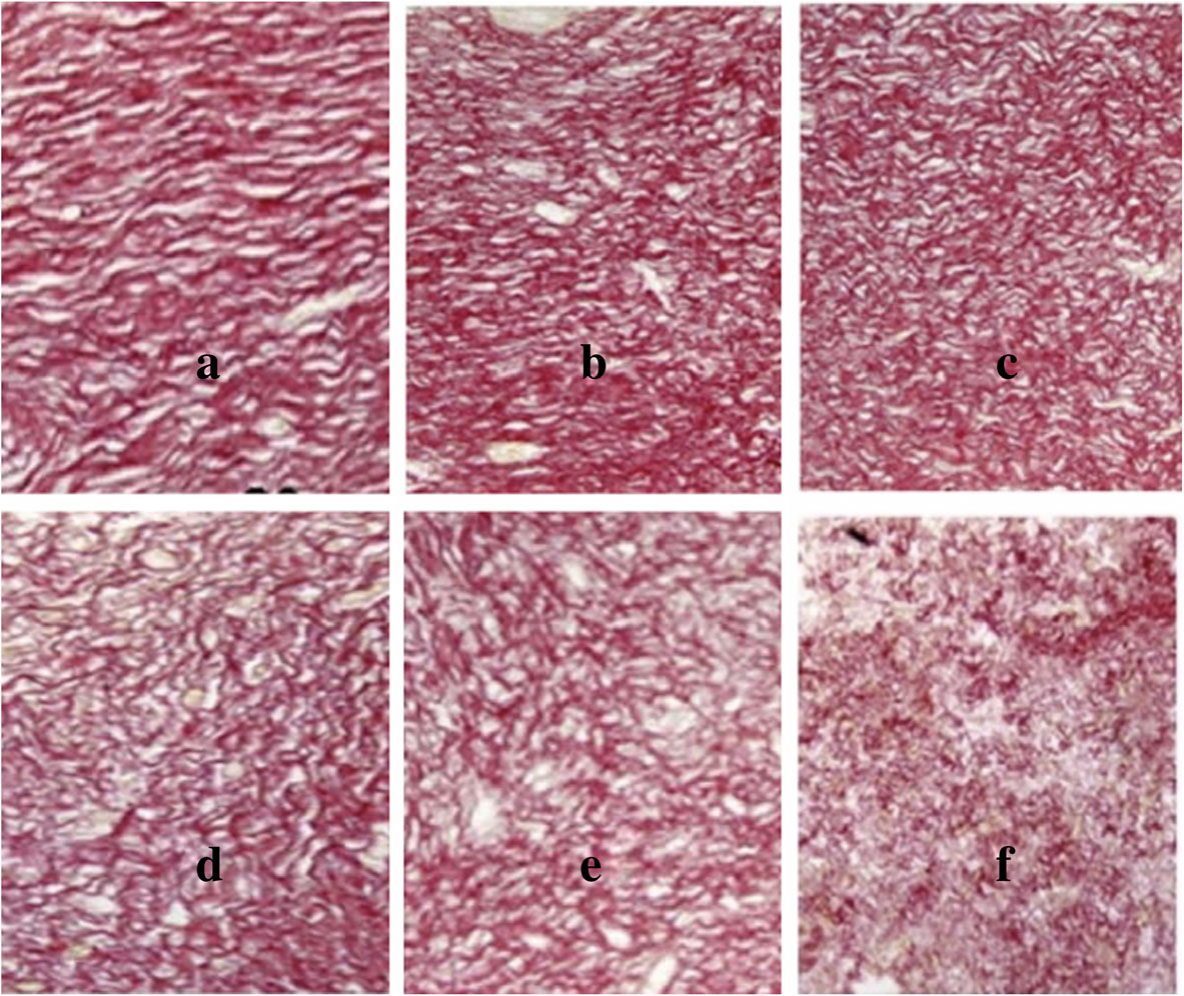
Effects of ivermetin cream on wound collagen content. The wound tissues were taken and fixed in 4% phosphate-buffered formaldehyde (PBF) and stained with Picrosirius Red solution. Photomicrographs showed the degree of red color intensity connoting collagen density as observed in Control (**a**), Ag S (**b**), 1% ivermectin (**c**), 0.30% ivermectin (**d**), 0.10% ivermectin (**e**), 0.03% ivermectin (**f**). Image Scale bar is 40 μm. Micrographs were captured at 400x magnification with a resolution of 12.0 Megapixels

Collagen Density Evaluation – The picrosirius red stain was used to detect collagen formation based on the intensity of the red colour of the stained fibres. Photomicrographs of Picrosirius-stained sections taken on day 21 showed decreasing collagen density with decreasing doses of ivermectin (Fig. 2). The percent collagen content quantified by wound densitometry on day 21 showed 72 ± 1.3, 59 ± 1.5, 58 ± 0.6, 57 ± 1.0, 56 ± 0.9, 54 ± 0.7 for control, Silver Sulfadiazine, ivermectin (1, 0.30, 0.10, 0.03% (w/w)) respectively in a decreasing trend of collagen content. This indicated that in the treatment groups, the lower doses of ivermectin did show lower collagen fibre density (****p < 0.0001) thus causing healing with minimal cicatrix formation.

### Effects of ivermectin on cutaneous wound biomarkers on day 7 and 21

**Fig. 3:**
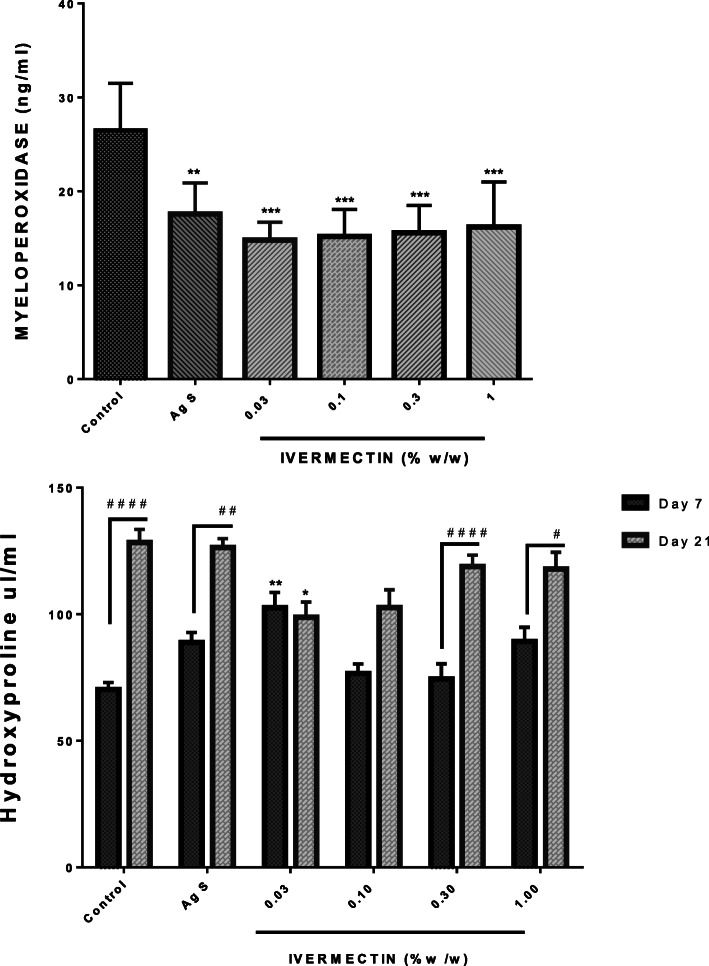
Effects of ivermectin cream on cutaneous wound tissue levels of MPO and hydroxyproline. Sprague-Dawley rats were anaesthetised and excision wounds created as described in the methods. Animals were sacrificed on either day 7 or day 21 post-wounding. The levels of myeloperoxidase on day 7 as well as levels of hydroxyproline on day 7 and day 21 were measured and presented as mean ± SEM. The statistical analysis for MPO levels was by One-Way ANOVA. * means *p* < 0.05, ** means *p* < 0.01,*** means *p* < 0.001 for treated groups when compared to the vehicle (naïve) control group. The statistical analysis for hydroxyproline was by Two-Way ANOVA followed by Bonferroni's post hoc test. * means *p* < 0.05,** means *p* < 0.01 when compared to the corresponding day control levels whilst # means *p* < 0.05, ## means *p* < 0.01, #### means *p* < 0.0001 when comparing levels on day 7 to day 21 within a group

Myeloperoxidase (MPO) levels - The MPO activity was studied to indirectly estimate the extent of neutrophil infiltration and activation at the wound site. Treatment with ivermectin cream (0.03–1% (w/w)) resulted in a statistically significant decrease in MPO activity. Silver Sulfadiazine used as the positive control exhibited similar activity as ivermectin. However, all the ivermectin doses were more effective in reducing MPO activity compared with the Silver Sulfadiazine. Figure 3 indicates receding MPO levels and hence neutrophil infiltration with ivermectin cream.

Hydroxyproline levels**-** Hydroxyproline levels were evaluated on day 7 and day 21. In all animals, hydroxyproline levels increased from Day 7 to Day 21. This increase in hydroxyproline was significant at high doses of ivermectin cream (0.3–1.0% (w/w)). Similarly, the Silver Sulfadiazine group and the non-treated groups also had significant increases in hydroxyproline on Day 7 and Day 21. However at lower doses of ivermectin (0.03–0.1% (w/w)), hydroxyproline levels did not increase significantly. The lower hydroxyproline levels were associated with lower doses of ivermectin-treated wounds on day 21 (Fig. 3).

### Effects of ivermectin on growth factors involved in wound healing on day 7 and 21

**Fig. 4:**
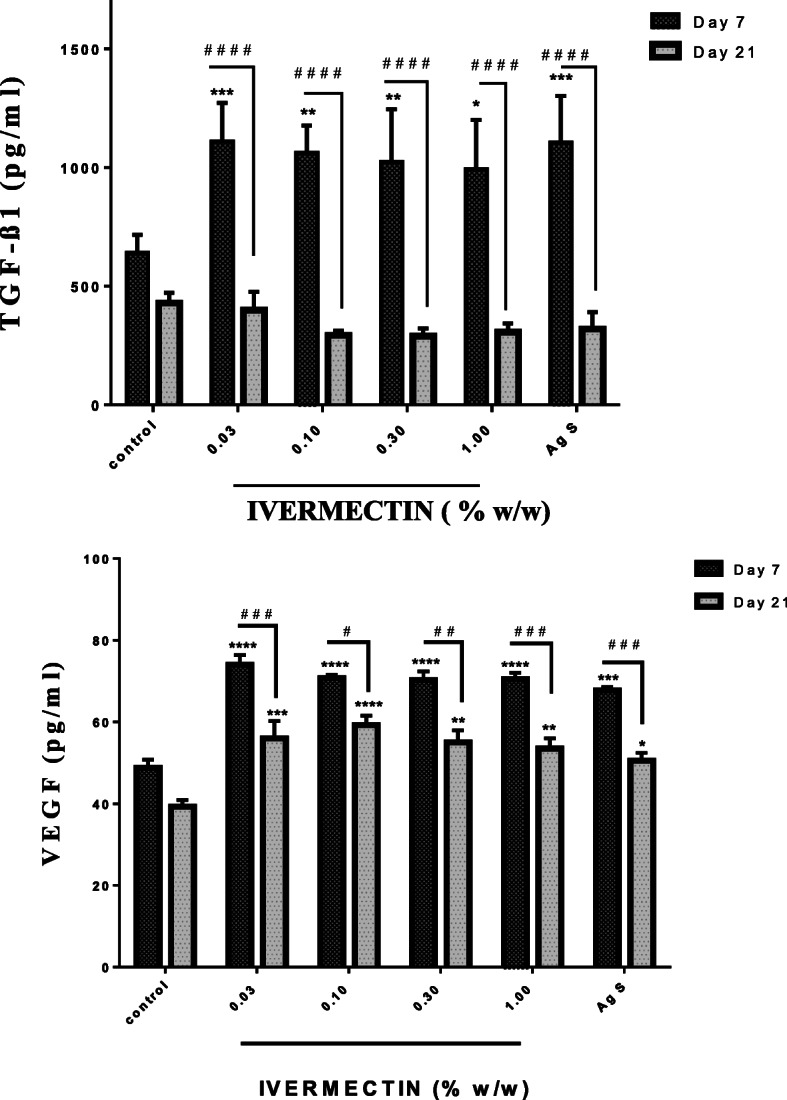
Effects of ivermectin cream on cutaneous wound levels of TGF-B1 and VEGF. Sprague Dawley rats were anaesthetised and excision wounds created as described in the methods. Animals was sacrificed either on day 7 or day 21 post-wounding. The levels of TGF-ß1 on day 7 and day 21 as well as the levels of VEGF on day 7 and day 21 were measured and presented as mean ± SEM. The statistical analysis was by Two-Way ANOVA. Results shown as *p < 0.05,**p < 0.01,***p < 0.001, ****p < 0.0001, for all treated groups compared to vehicle (naïve) control group and # *p* < 0.05, ## *p* < 0.01, ### *p* < 0.001, #### *p* < 0.0001 represent comparison between levels on Day 7 and 21 within a group

TGF-β1 - Treating wounds with ivermectin cream (0.03–1% (w/w)) resulted in significant increases in the levels of TGF-β1 within the first 7 days of treatment. The low dose of ivermectin was more effective in increasing levels of TGF-β1 than the highest dose of 1%. The positive control had similar effects as ivermectin in this study. Levels of TGF-β1 decreased in all treated groups from Day 7 to 21. On day 21, there was no difference between ivermectin treated groups and non-treated groups. (Fig. 4).

VEGF Levels- Similarly, the levels of VEGF were significantly higher in ivermectin treated groups than non-treated groups on day 7. All doses of ivermectin were more effective at increasing the levels of VEGF than Silver Sulphadiazine. Although the levels reduced from day 7 to 21, the levels of VEGF in ivermectin treated wounds were still significantly higher than that of the control group (Fig. 4).

### Effects of ivermectin on cytokines in cutaneous wounds on day 7 and 21

#### IL-1α, IL-4, IL-10 and TNF-α levels

**Fig. 5:**
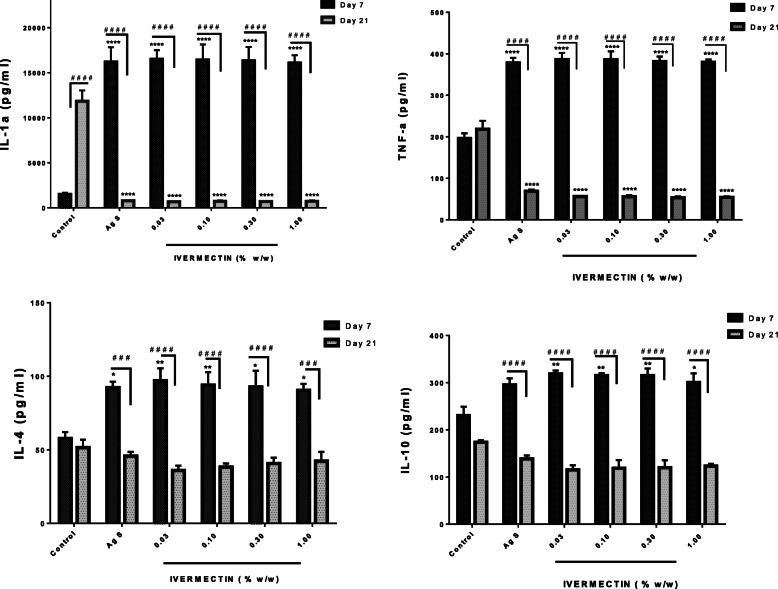
Effects of ivermectin on IL-1α, IL-4, IL-10, and TNF-α levels in cutaneous wounds. Sprague Dawley rats were anaesthetised and excision wounds created as described in the methods. Animals were sacrificed either on day 7 or day 21 post-wounding. The levels of the cytokines on day 7 and day 21 are presented graphically as mean ± SEM. The statistical analysis is by two-Way ANOVA followed by Bonferrroni's post hoc test. Results presented as *p < 0.05, **p < 0.01, ***p < 0.001, ****p < 0.0001 when comapred to the vehicle (naïve) control group and # *p* < 0.05, ## *p* < 0.01, ### *p* < 0.001, #### *p* < 0.0001 represent comparison between levels on Day 7 and 21 within a group

The treatment of cutaneous wounds with ivermectin cream (0.03–1%) resulted in a significant increase in IL-1α and TNF- α levels on day 7 when compared to control. The spike in these cytokines on day 7 was reduced to levels significantly lower than that of untreated controls on day 21. All the doses of ivermectin were equally effective in altering IL-1α and TNF- α levels on day 7 and drastically reducing it by day 21. The control levels of both cytokines increased on day 21 when compared to day 7 (Fig. 5). Similar trend was seen with the levels of IL-4 and IL-10 on days 7 and 21. However, the control levels of IL-4 and IL-10 decreased on day 21 compared to day 7 but was not statisitcally significant.

### Effects of Ivermectin on eicosanoids involved in wound repair on day 7 and 21

**Fig. 6:**
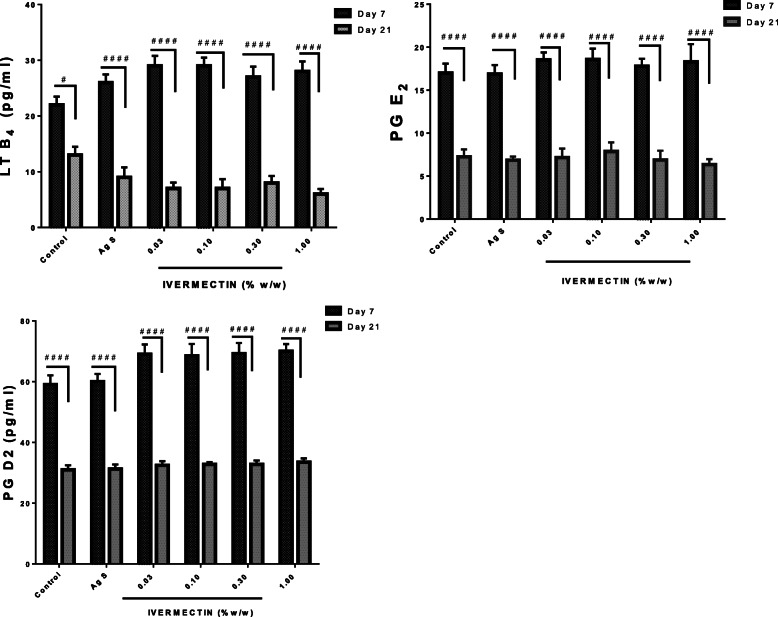
Effects of ivermectin on LTB_4_, PGE_2_ and PGD_2_ levels in cutaneous wound. Sprague Dawley rats were anaesthetised and excision wounds created as described in the methods. Animals were sacrificed either on day 7 or day 21 post-wounding. The levels of eicosanoids on day 7 and day 21 are presented graphically as as mean ± SEM. The Two-Way ANOVA statistical analysis results are shown as; # *p*< 0.05, #### *p*< 0.0001 when levels on day 7 are compared to levels on day 21 of the same group

In all experimental animals, levels of LTB_4_, PGD_2_, PGE_2_ levels increased on day 7 but reduced on day 21. Although not statistically significant, the levels of increases recorded in the ivermectin group (0.03–1%) were higher than the levels in the non-treated controls (Fig. 6).

## Discussion

A wound, which is a break in the structural integrity or continuity **o**f a tissue or organ, may not only compromise its physiological and biological function but can provide favorable conditions for microbial survival [1, 3]. Optimal wound healing has become the main objective in clinical settings. Researches across the globe in this domain aim at finding therapeutic agents which can speed up wound healing through all its stages to avoid any possible delays due to comorbidities such as immunosuppressive pathologies, diabetes, cardiovascular diseases, hyperlipidemias, clotting disorders, etc. [4, 15]. Ivermectin has been used in the treatment of parasite-infected wounds in medicine. The main objective of this study was to determine whether ivermectin, a well-known antiparasitic agent, exhibits wound healing property and elucidate the mechanisms involved [16].

The skin irritation test was carried out to cogently choose doses of ivermectin that would cause no lesion or toxicity. The Primary Irritation Index for ivermectin was very low (PII < 1) and its LD_50_ was estimated to be above 10% (w/w). The results corroborate with previous studies [17, 18]. Subsequently, doses below 10% (w/w) were selected for all other experiments, both morphologic and molecular. The choice of 1% (w/w) as the highest dose in all the experiments was informed by its safety based on the dermatotoxicity test results and also supported by clinical evidence of effectiveness in treating inflammatory conditions [16].

The macroscopic wound healing indices were indicative that optimal wound healing progression by ivermectin is from the lowest to the highest dose. In the study, low doses of ivermectin showed lower signs of toxicity but were equally efficacious as evidenced in the indices such as exudation, edge edema, hyperemia, and granulation tissue. Upon damage, keratinocytes release VEGF and granulocyte-macrophage colony stimulating factor, early response genes, which promote recruitment and proliferation of macrophages, fibroblast, and keratinocytes by modulating cytokines and growth factors [19, 20]. Wound exudation, edema, hyperemia at the early stages are partly due to the leucocyte infiltration into the wounded area. The ability of ivermectin to suppress macroscopic wound healing indices by modulating the acute inflammatory phase may be a confirmation of its purported anti-inflammatory activity. However, the lack of dose-dependency in the effects indicate the possibility of other mechanisms, especially its anti-bacterial activity, contributing to the observed wounding healing effect [21, 22].

Furthermore, the “time to heal” and the percent wound contraction for lower doses of ivermectin showed that they were at least as effective as the higher doses. Morphological evaluation of the wounds at different stages of the healing process indicated minimal scarring and greater restoration with hair re-growth at the lower doses; as such 0.03% (w/w) ivermectin-treated wounds had minimal scarring as compared to the 1% (w/w) ivermectin-treated wounds. Indeed, the minimal scarring and the hair re-growth gave the 0.03% (w/w) ivermectin-treated wounds a higher esthetic effect compared to the higher doses. This difference may be related to ivermectin reducing or altering the formation and/or deposition of collagen fibers. Other agents have been reported to exhibit similar effects [15, 23]. It is also possible that the minimal scarring seen at lower doses of the cream may be partly due to increased wound neo-vascularization induced by ivermectin as well [24]. Furthermore, ivermectin has shown to increase and decrease levels of hydroxyproline on day 7 and day 21 respectively compared to control. Ivermectin’s effects on hydroxyproline turnover on day 21 correlatewell with collagen deposition in wounds measured in this study.

The inflammatory response that erupts during wounding is very important. However, intense and prolonged inflammation can be detrimental to wound healing [24, 25]. Ivermectin, as shown in this study, modulate levels of MPO possibly through altering neutrophil infiltration [8]. Cytokines in this study, both pro-inflammatory such as IL-1α, IL-4, and TNF-α and anti-inflammatory IL-10 are very important and crucial in the wound healing process. They impact various stages of wound healing via cell stimulation, protein metabolism, chemoattractant effect, regulatory activity on immunological responses and modulation of inflammation aiming at a balanced healing outcome [8, 26]. Eicosanoids such as PGD_2_, PGE_2_ and LTB_4_ are reported to participate in wound healing and can be released at contrasting stages of the healing process, playing an important function in the initiation and the resolution of inflammation [22, 27, 28]. The results showed that ivermectin modulated levels of the cytokines (IL-1α, IL-4, IL-10, and TNF-α) and eicosanoids (PGD_2_, PDE_2_ and LTB_4_) based on the stage of inflammation and the phase of wound healing by enhancing the release of anti-inflammatory cytokine (IL-10) and PGD_2_ whilst limiting the release of the pro-inflammatory cytokines (IL-1α, IL-4 and TNF-α) and lipid mediators (PDE_2_, LTB_4_). This supports previous studies [26, 29], suggesting that ivermectin possibly balances the intensity and extent of the inflammation probably through a systematic adjustment of phospholipid metabolism from pro-inflammatory to anti-inflammatory eicosanoids

The TGF-β1 at the site of injury serves as a chemo-attractant for Fibroblast Growth Factor (FGF), and TNF-α secreting macrophages. It also modulates collagen deposition, the expression of collagenase and enhances smooth muscle cell proliferation [8]. Ivermectin's ability to modulate the activity of TGF-β1 is confirmed by its modulatory effect on the pro-inflammatory cytokines (IL-1α and TNF-α) and the decreased collagen density on day 21. As noted in the study on day 21 and also reported by other authors, the transition of the healing from the initial stage to the terminal stages is characterized by decreased expression TGF-β1 [30]. More importantly, the levels of TGF-β1 may also explain why low doses of ivermectin healed wounds with minimal scarring as TGF-β1 affects collagen deposition. Low doses of ivermectin can potentially be experimented in cosmetology for scarless skin wound healing from burns, major trauma, and surgeries.

The VEGF is another indispensable biological marker in wound healing. Its significance emerges from its angiogenic and proliferative function in epithelialization and collagen deposition [31]. Even though VEGF and TNF-α are simultaneously released by activated platelets and macrophages during wounding, the activities of VEGF in the wound area are also dependent on TNF-α, as the latter induces the expression of the former in fibroblast and keratinocytes [5]. TGF-B1 induces the secretion of TNF-α by macrophages and the expression of VEGF, thereby affecting the release of VEGF in manifold [6, 7]. Ivermectin in this study modulated the activity of VEGF by keeping its levels optimally low at day 21 and hence regulating angiogenesis. This is probably due to the modulatory effects of ivermectin on both TNF-α and TGF-β1 observed as well as its direct impact on VEGF activity.

## Conclusion

Ivermectin promotes cutaneous wound healing through modulation of inflammation and regulation of TGF-β1 and VEGF levels. Low doses of ivermectin cream have the potential to be used in treating wounds with minimal scar tissue formation.

## Methods

### Animals

Male Sprague Dawley rats (*n* = 100, wt. = 180 ± 5 g, age = 8 weeks old, Strain Code: 400) were purchased from Noguchi Memorial Institute for Medical Research (NMRI), University of Ghana, Legon, and kept in the Animal House of the Department of Pharmacology, KNUST. They were housed in groups of six (6) in stainless steel cages (34x47x18 cm^3^). The rats were fed with normal pellet diet commercial chow from AGRICARE LIMITED, Kumasi, Ghana. They were given water ad libitum and maintained in a 12-h light-dark cycle with softwood shavings as bedding and a temperature of 25 °C (±2^o^). All procedures and techniques used in the various experiments were in accordance with the Guide for the Care and Use of Laboratory Animals (Institute for Laboratory Animal Research, 2016). The Faculty of Pharmacy and Pharmaceutical Sciences, College of Health Sciences, KNUST Ethical Review Committee approved all protocols, for this work (Ref no: PH/ETH/097/0118).

### Chemicals

Ketamine HCl (50 mg/ml, Rotexmedica GmbH, Germany), xylazine HCl (100 mg/mL, Bayer, Leverkusen, Germany). Dulbecco’s Phosphate Buffered Solution (Life Technologies, Germany). Picro Sirius Red Stain Kit (Cambridge, MA, USA). Ivermectin powder USP (Letco Medical, LLC), Silver Sulfadiazine (1% (w/w), Pharmacia, Germany). ELISA kits for TNF-α, IL-1α, IL-1β, IL-10, IL-4 (Abcam, USA), TGF-β_1_, VEGF (RayBiotech, GA, US), LTB4, PGD_2_ & PGE_2_ (Cayman Chemical, MI - USA).

### Acute dermatotoxicity study

The degree of dermal irritation was determined in rats using the occluded dermal irritation test as described in previous works [32]. Sprague Dawley rats (*n* = 9) were used for this test and each animal served as test and control. On the first day (Day 0) of the test period, the fur was clipped from the back (about 12% of the total body surface area) of each rat. The left side (about 6 ± 1.5 cm^2^) served as a test site, while the right side as a control site. Rats were caged separately and left unperturbed for 24 h. On Day 1 of the test period, three selected doses of ivermectin cream (1, 3, 10% (w/w)) were evenly applied to the left shaved area and the vehicle of the cream was also applied to the right side to serve as the control. The skin was covered with gauze and a non-irritating adhesive plaster. After 24 h of exposure, the coverings were removed and the test site was cleaned with normal saline-soaked gauze. The experimental animals were examined for the presence of erythema/redness and edema/swelling according to the dermal irritation scoring system and the Primary Irritation Index estimated [33].

### Cutaneous wound contraction study

Thirty male Sprague-Dawley rat (*n* = 30, wt. = 180 ± 5 g) were anesthetized with Ketamine (80 mg/kg) + Xylazine (15 mg/kg) *IP*. The back of each rat was shaved with care to avoid traumatizing skin before procedural wounding. The shaved backs were thoroughly wiped with 70% Ethanol. An incision on the skin about 500 mm^2^ was carefully created using scissors. At the end of the excisional wounding exercise, rats were returned to their cages per their groupings as modeled by Frank and Kampfer (2003) [34]. For the study, Sprague-Dawley rats were randomly assigned into 6 groups (*n* = 5) and groups treated as follows;

Group 1 = (Naive control group) where only petroleum jelly was applied,

Group 2 = (Positive control) Silver Sulfadiazine 1% (w/w) treated group,

Groups (3–6) = (Test groups) treated with 1, 0.3, 0.1, 0.03% (w/w) ivermectin cream respectively.

### Assessment of macroscopic wound healing indices

The wounds were evaluated in vivo on day 0, 2, 5, 7, 9, day 13, 15, and on day 21 for cutaneous wound healing indices such as Exudation, Edge edema; Hyperemia, and Granulation Tissue deposition based on methods described by Gupta et al. (2011) [35] and subsequently modified by Eyarefe et al.(2017 )[36]. Wound Exudation, Wound Edge Edema; Wound Hyperemia was scored by two blinded pathologists working independently as 0 = Absent, 1–2 = Mild, 3–4 = Moderate, 5–6 = Severe. Similarly, Granulation Tissue was graded (0 = Absent, 1–2 = Low, 3–4 = Moderate, 5–6 = High).

### Wound morphometry: evaluation of cutaneous wound contraction

The cutaneous wound analysis was carried out on wound microphotographs taken on days 0, 2, 5, 7, 9, 13, 15, and day 21 post-wounding to assess the progression of wound healing. This was done with the aid of Imito Measure® Application (Imito AG, Flüelastrasse, Zürich, Switzerland) and the Java-based Image Processing and Analysis software, ImageJ Software (National Institutes of Health, USA). The time-course of cutaneous wound healing was determined as the wound contraction or closure rate using the formula [36]:

$$ \mathrm{WC}\%=\left[\frac{\left(\mathrm{WA}\ \mathrm{at}\ \mathrm{d}0-\mathrm{WA}\ \mathrm{at}\ \mathrm{d}\mathrm{n}\right)}{\mathrm{WA}\ \mathrm{at}\ \mathrm{d}0\ }\right]\mathrm{x}\ 100 $$

where WC = Wound Contraction, WA = Wound Area, d_0_ = Day 0 and d_n_ = Day n or any other day.

### Histopathological analysis on day 21

Animals were euthanized with ketamine–xylazine (100 μL of a 10:1 ketamine–xylazine solution) followed by cervical dislocation on day 21 post-wounding. The wounds and their edges were harvested under sterile conditions. The wound tissues were taken at the final time-point (day 21) post-excision and fixed in 4% phosphate-buffered formaldehyde (PBF). The specimens were processed for histopathology. They were embedded in paraffin wax and serialized sections of 5 μm of block thickness were mounted on glass slides. The sectioned specimens were dewaxed and serially rehydrated with distilled water. The blocks were stained with Picrosirius Red solution prepared by saturating Sirius Red solution in an aqueous picric acid solution as described by Bitencourt et al. (2011) [37]. The slides were carefully examined using a light microscope (DMC 5400 Colour CMOS Camera fitted to a Light Microscope (Leica DM 2500) with LAS Software 2017 version). Micrographs were captured at 400x magnification at a resolution of 12.0 megapixels. Photomicrographs of Picrosirius red-stained tissues were used for quantifying the collagen content in the wound tissues [38, 39]. The collagen quantification analysis relating to the fiber-dense area of the micrographs was carried out by digital densitometry recognition and the areas were recorded as percentiles of the total area of the field using the Java-based Imaging software, Image J (National Institutes of Health, USA).

### Ivermectin on cutaneous wound histochemistry on day 7 and 21

Cutaneous excision wounds were created in six (6) experimental groups of Sprague-Dawley rats (*n* = 10). The groups were treated as follows: Group 1 = (Naive control group) where only petroleum jelly was applied, Group 2 = (Positive control) were treated with Silver Sulfadiazine 1% (w/w), Groups (3–6) = (Test groups) were treated with 1, 0.3, 0.1, 0.03% (w/w) ivermectin respectively. Five rats from each group were euthanized on day 7 and day 21 post-wounding. The wounds and their edges were harvested by sterile cut using forceps and scissors. The wound tissues were fixed in 4% PBF and were processed in compliance with histochemical protocols. The wound tissues were then homogenized (Tetra Pak® Homogenizer, Tetra Laval, Switzerland). The samples were centrifuged at 1500×g and kept at − 80 °C until the time of assay. The biomarkers (MPO, hydroxyproline), growth factors (TGF-β1, VEGF), cytokines (IL-1α, IL-4, IL-10, TNF-α), and eicosanoids (PGE_2_, PGD_2_, LTB_4_) were assayed using their respective ELISA kits and quantified based on manufacturers’ protocols.

### Statistical analysis

Results were presented as mean ± SEM or as percentiles where appropriate. The statistical tests and analyses of various experiments in this work were performed using GraphPad version 8.0 (San Diego, California, USA). Statistical analysis of dose and effect among group means were done using one-way ANOVA. Dose, effect with time was analyzed using two-way ANOVA followed by Bonferroni’s post hoc test. The *p*-values less than 0.05 (*p* < 0.05) were indicative of statistical significance.

## Supplementary information

**Additional file 1: Figure S1.** Morphometric evaluation of Ivermectin effects on cutaneous wound contraction. Sprague Dawley rats were anaesthetised and excisional wounds and created as described in methods. Digital photographs were taken during the wound healing experiment and those at three critical time-points day 2, 7 and 21.

## Data Availability

The datasets used and/or analysed during the current study are available from the corresponding author on reasonable request.

## Abbreviations

TGF-β1: Transforming Growth Factor-Beta 1
VEGF: Vascular Endothelial Growth Factor
IL: Interleukin
TNF-α: Tumor Necrosis Factor Alpha
PG: Prostaglandins
LT: Leaucotriene
PII: Primary Irritation Index
MPO: Myeloperoxidase
PBS: Dulbecco’s Phosphate Buffered
Ag S: Silver Sulfadiazine
