# Supplementary material for: Mechanisms of ivermectin-induced wound healing
Source: BMC Vet Res. 2020 Oct 20;16:397. doi: 10.1186/s12917-020-02612-z (PMC7576857; doi:10.1186/s12917-020-02612-z)
Supplement: Supplementary file 1 — Additional file 1: Figure S1. Morphometric evaluation of Ivermectin effects on cutaneous wound contraction. Sprague Dawley rats were anaesthetised and excisional wounds and created as described in methods. Digital photographs were taken during the wound healing experiment and those at three critical time-points day 2, 7 and 21. [file 12917_2020_2612_MOESM1_ESM.docx]

Control SSD 1% 0.30%. 0.10% 0.03%

Ivermectin

Ivermectin


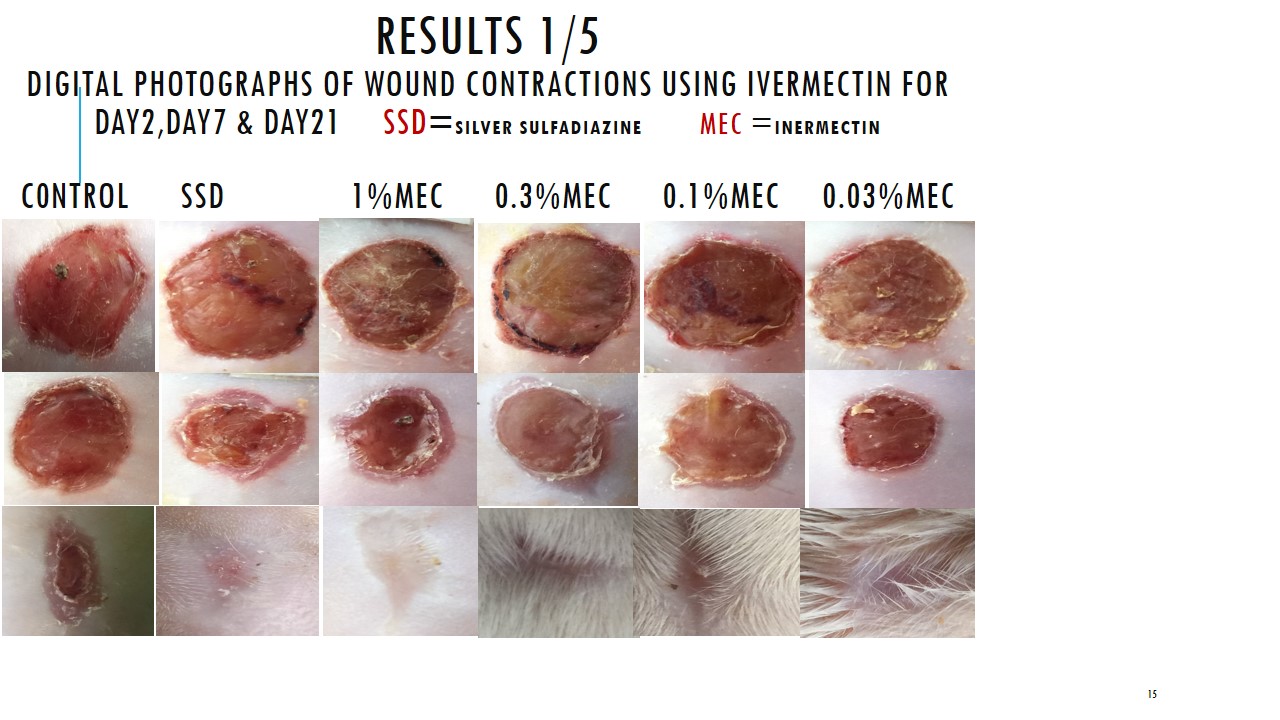


Day 2

 Day 7

Day 21

**Figure S1. Morphometric evaluation of Ivermectin effects on cutaneous wound contraction.** Sprague Dawley rats were anaesthetised and excisional wounds and created as described in methods. Digital photographs were taken during the wound healing experiment and those at three critical time-points day 2, 7 and 21
